# Safety and efficacy of aspirin and indobufen in the treatment of coronary heart disease: a systematic review and meta-analysis

**DOI:** 10.3389/fcvm.2024.1412944

**Published:** 2024-08-15

**Authors:** Xiaochen Zhang, Qiaoyan Yan, Jiao Jiang, Hua Luo, Yu Ren

**Affiliations:** ^1^ Department of Psychiatry, Taizhou Second People’s Hospital, Taizhou, Zhejiang, China; ^2^ Department of Pharmacy, Taizhou Hospital of Zhejiang Province Affiliated to Wenzhou Medical University, Taizhou, Zhejiang, China; ^3^ Department of Orthopedics, Taizhou Hospital of Zhejiang Province Affiliated to Wenzhou Medical University, Taizhou, Zhejiang, China

**Keywords:** aspirin, indobufen, coronary heart disease, efficacy, meta-analysis

## Abstract

**Purpose:**

This meta-analysis aimed to compare the safety and efficacy of aspirin and indobufen in patients with coronary heart disease. The primary focus was on the incidence of cardiovascular events, bleeding events, and gastrointestinal reactions. Given the relatively limited research on indobufen, this study utilized aspirin as a control drug and employed meta-analysis to integrate existing clinical studies. The goal was to provide a reference for the clinical use of indobufen and to suggest directions for further largescale, multicenter prospective studies.

**Methods:**

This review adhered to the Preferred Reporting Items for Systematic Reviews and Meta-Analyses (PRISMA) guidelines. We conducted a comprehensive search of the PubMed, EMBASE, WOS, and Cochrane Library databases to identify all relevant literature on indobufen. A total of nine trials met the inclusion criteria, encompassing seven randomized controlled trails (RCTs) and two retrospective studies. Categorical variables were analyzed using odds ratio and random effects models.

**Results:**

The meta-analysis included nine trials, comprising seven RCTs and two retrospective studies. The pooled results indicated that indobufen significantly reduced the incidence of minor bleeding events, and gastrointestinal discomfort compared to aspirin. However, both drugs had similar effects on the incidence of recurrent angina pectoris, myocardial infarction and mortality due to coronary heart disease.

**Conclusion:**

Indobufen was associated with fewer gastrointestinal reactions and a low risk of bleeding, making it a viable option for patients with high-risk factors for bleeding and gastric ulcers. Despite this, indobufen's short history and limited evidence base compared to aspirin highlight the need for further research. Aspirin remains widely available, cost-effective, and the preferred drug for the primary and secondary prevention of cardiovascular and cerebrovascular diseases. Indobufen or other antiplatelet agents should only be considered when aspirin is not tolerated or contraindicated. Further clinical trials are necessary to determine whether indobufen can replace aspirin.

**Systematic Review Registration:**

https://www.crd.york.ac.uk/, identifier [CRD42024523477].

## Background

The activation and aggregation of platelets are crucial processes in the pathogenesis of atherosclerosis thrombosis (1). Therefore, antiplatelet therapy is a cornerstone in the treatment of coronary heart disease. Indobufen and aspirin are both inhibitors of platelet cyclooxygenase (COX), reducing the production of thromboxane A2, a potent promoter of platelet aggregation. Despite their similar roles in inhibiting platelet aggregation and reducing thrombosis, they differ significantly in their mechanisms of action. Indobufen and aspirin are both inhibitors of platelet cyclooxygenase (COX), reducing the production of thromboxane A2, a potent promoter of platelet aggregation. Despite their similar roles in inhibiting platelet aggregation and reducing thrombosis, they differ significantly in their mechanisms of action.

Indobufen offers several advantages over aspirin due to its high selectivity for inhibiting platelet COX-1. Unlike aspirin, which has irreversible effects, the inhibitory effects of indobufen on platelet function are reversible and return to baseline within 24 h after discontinuation. Additionally, indobufen does not significantly impact prostacyclin, posing a lower risk of gastrointestinal injury, bleeding, and renal damage. It also does not affect uric acid metabolism. Indobufen's multi-target approach inhibits platelet aggregation induced by various factors, including ADP, AA, collagen, epinephrine, and platelet factor, enhancing its efficacy in overcoming drug resistance.

Consequently, guidelines and textbooks recommend indobufen as an alternative to aspirin for patients at a high risk of bleeding and those with gastric ulcers (2, 3). While aspirin's first-line status in preventing and treating cardiovascular and cerebrovascular diseases is well-supported by extensive evidence-based research, the evidence for indobufen remains comparatively limited.

This article systematically reviewed clinical trials and cohort studies on the efficacy and safety of indobufen in treating of coronary heart disease, usingaspirin as the control. By employing meta-analysis, we aimed to integrate existing clinical studies to provide a reference for the clinical use of indobufen and to underscore the need for further large-sample prospective research.

## Methods

According to the PRISMA (Preferred Reporting Items for Systematic Reviews and Meta-Analyses) statement, this meta-analysis was performed in agreement (4). The protocol for this meta-analysis was registered on PROSPERO (Registration No: CRD42024523477).

### Inclusion criteria

Study type: randomized controlled trial (RCT), cohort study or case-control study. Study population: patients with coronary heart disease. Intervention and control: Indobufen was used in the treatment group, and aspirin was used in the control group. Outcome index: adverse cardiovascular events (ACE) including recurrent angina, nonfatal myocardial infarction, and cardiac death, bleeding events [according Bleeding Academic Research Consortium (BARC) (5)] including minor bleeding (BARC grades 0–2), major bleeding (BARC grades 3–5) and any bleeding (BARC grade 0–5), and gastrointestinal adverse reactions.

### Exclusion criteria

Letters, case reports, meetings, reviews, animal trials, or republished studies; indobufen was not used in the treatment group; Studies lacking a control group; Patients receiving oral or intravenous anticoagulant therapy for another condition, such as atrial fibrillation, pulmonary embolism, lower limb venous thrombosis, or an artificial heart valve.

### Search strategy

One of the authors performed the search in PubMed, EMBASE, Web of Science, and the Cochrane Central Register of Controlled Trials from the inception dates to March 21, 2024, using the keywords “Indobufen” AND “Aspirin” AND “Coronary Diseases”. The detail of searching strategy for each database was shown in Supplementary Table S1.

### Study selection

Two researchers (YR and XCZ) screened the retrieved literature strictly against inclusion and exclusion criteria. First, the documents that meet the inclusion criteria are read in full by reading the title and abstract, and the included papers are finally confirmed. If two researchers do not agree during the literature screening process, it will be left to the senior researcher (HL).

### Data collection process

Data on relevant outcome measures that met the inclusion criteria were extracted from the literature, including author year, study design type, country, sample size, participants, TXA treatment, age, outcomes, etc.

### Assessment of risk of bias and quality of evidence

Two researchers (QYY and YR) independently assessed the quality of all included trials based on Cochrane risk-of-bias criteria (6). The ROBINS-I tool evaluates bias risk for non-RCT across domains like confounding, selection, intervention classification, deviations, missing data, outcome measurement, and result reporting.

### Data synthesis

The Meta-analysis was performed using Stata (version 17; StataCorp, 2021) software. The heterogeneity was assessed by using the Q test and *I*^2^ value calculation. If the heterogeneity was absent (*P* > 0.1 and *I*^2^ < 50%), the data was combined with a fixed effect model. The random effects model was used if the heterogeneity was present (*P* < 0.1 or *I*^2^ > 50%). The odds ratio (OR) and their associated 95% confidence interval (CI) were used to assess outcomes for dichotomous outcomes. Continuous outcomes were analyzed using mean, SD, and sample size to provide a mean difference (MD) between the indobufen and aspirin groups. A *P* value less than 0.05 suggested that the difference was statistically significant.

### Sensitivity analyses

We performed a sensitivity analysis by excluding the largest trial, cluster randomized or quasi-randomized trials, and trials with a high risk of bias, using random effect models.

## Results

**Figure 1:**
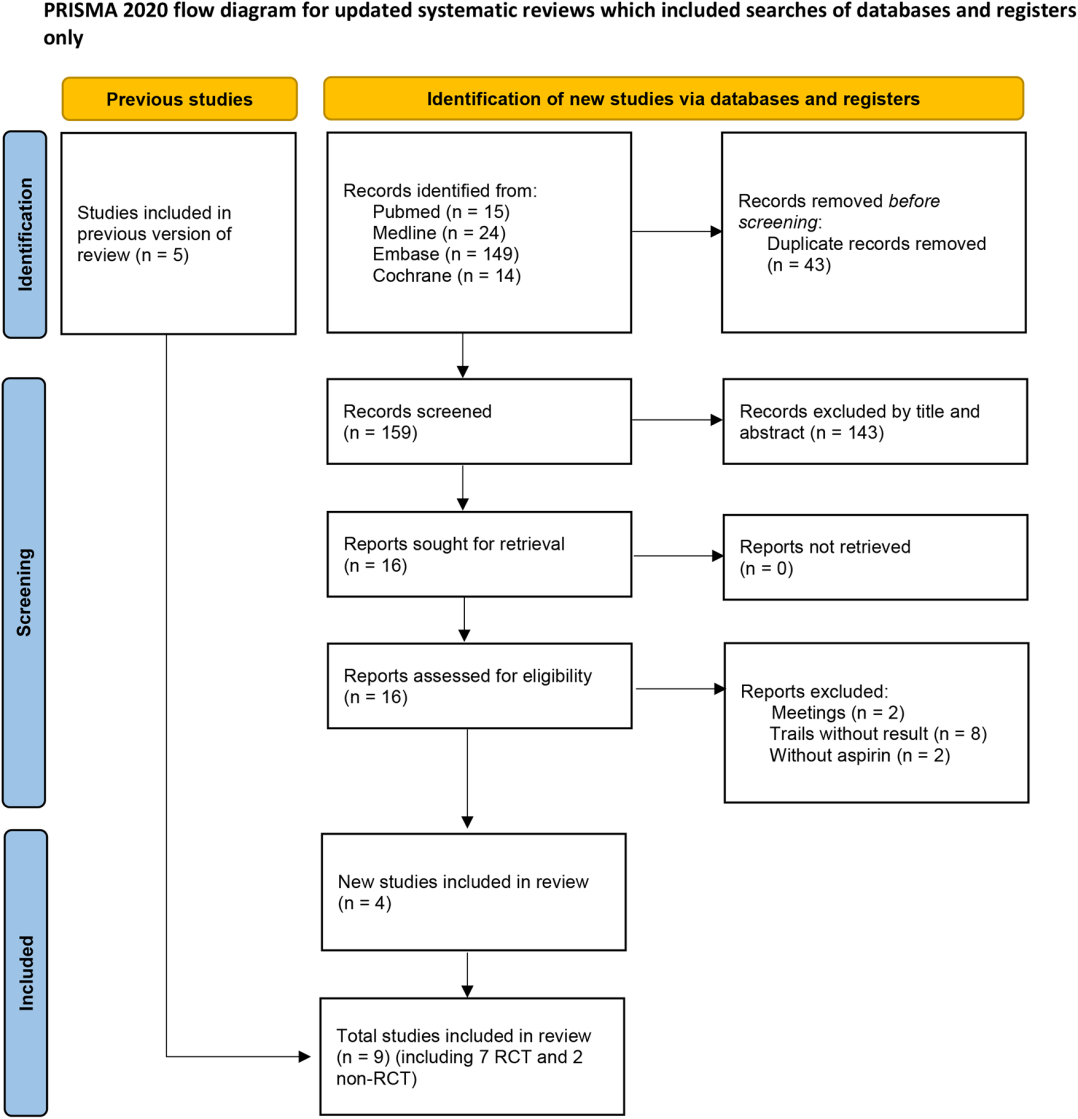
Flow diagram for search and selection of included studies.

After screening 202 studies, 43 duplicates were removed, and 143 irrelevant studies were excluded based on titles and abstracts. The full text of 16 articles were reviewed, resulting in the elimination of 12 articles. Among these, eight trials lacked results (7–13), two were conference abstract (14, 15), and two studies used dipyridamole in addition to aspirin in the control group (16, 17), preventing extraction of data specific to aspirin. Five studies were already included in previous meta-analysis. Ultimately, nine trials, comprising seven randomized controlled trials (RCTs) and two retrospective studies were included in this meta-analysis. The literature screening process is illustrated in Figure 1, and the essential characteristics of the included studies are detailed in Table 1.

**Table 1:** Characteristics of included studies.

Study	Country	Participants	Treatment	Design	Age		Sex (F/M)		Outcomes	No. of subject	
					Aspirin	Indobufen	Aspirin	Indobufen		Aspirin	Indobufen
Bai et al. (18)	China	Patients after CABG	100 mg aspirin qd + 75 mg clopidogrel qd. 100 mg indobufen qd + 75 mg clopidogrel qd.	RCS	59.7 ± 7.2	60.3 ± 6.6	18/57	14/62	Adverse cardiovascular event, gastrointestinal bleeding	76	76
Wu et al. (19)	China	Coronary artery disease with symptoms of angina pectoris or documented ischemia	100 mg aspirin qd + 75 mg clopidogrel qd. 100 mg indobufen bid + 75 mg clopidogrel qd.	RCT	61.2 ± 8.4	61.0 ± 8.3	846/1,447	737/1,521	Cardiovascular death, nonfatal MI, ischemic stroke, ST, BARC type bleeding;	2,293	2,258
Shi et al. (14)	China	Stable coronary heart disease after PCI	aspirin 100 mg qd + clopidogrel 75 mg qd indobufen 100 mg bid + clopidogrel 75 mg qd indobufen 100 mg bid, aspirin 100 mg qd	open label crossover study	63.07 ± 6.46	2/54	AA-PAR, ADP-PA, PRI-VASP, plasma/Urinary TXB2, Gastrointestinal adverse reactions, BARC type bleeding;	56			
Yang et al. (15)	China	Patients with coronary atherosclerosis	asprini 100 mg qd; indobufen 100 mg bid	RCT	60.8 ± 10.7	60.7 ± 8.0	13/17	11/21	PLAA, plasma TXB2, urinary 11-dh-TXB2, gastrointestinal adverse reactions, BARC type bleeding	30	32
Chen (20)	China	Patients with unstable angina pectoris	100 mg aspirin qd + 75 mg clopidogrel qd. 100 mg indobufen bid + 75 mg clopidogrel qd.	RCT	69.46 ± 5.72	68.32 ± 4.72	35/40	39/36	Clinical effect, bleeding events (oral bleeding)	75	75
Zhang (13)	China	ACS	100 mg aspirin qd + 75 mg clopidogrel qd. 100 mg indobufen bid + 75 mg clopidogrel qd.	RCT	61.2 ± 7.4	62.2 ± 6.7	14/21	13/22	Adverse cardiovascular events, bleeding events (defecate occult blood, ecchymosis)	35	35
Zhou (21)	China	NSTE-ACS	100 mg aspirin qd + 75 mg clopidogrel qd. 100 mg indobufen bid + 75 mg clopidogrel qd.	RCT	68.5	67.3	14/16	13/17	Adverse cardiovascular events, gastrointestinal adverse reactions, bleeding event (bleeding gums, nasal bleeding)	30	30
Wen (23)	China	PCI	100 mg aspirin qd + 75 mg clopidogrel qd. 100 mg indobufen bid + 75 mg clopidogrel qd.	RCT	57 ± 9	58 ± 7	8/24	6/26	Adverse cardiovascular event, gastrointestinal adverse reactions, bleeding event (bleeding gums,)	32	32
Lv et al. (22)	China	Unstable angina pectoris	100 mg aspirin qd + 75 mg clopidogrel qd. 100 mg indobufen bid + 75 mg clopidogrel qd.	RCT	62.49 ± 4.35	61.61 ± 4.58	38/57	41/54	Gastrointestinal adverse reactions, bleeding event (occult blood in stool)	95	95

CABG, Coronary artery bypass grafting; PCI, percutaneous transluminal coronary intervention; ACS, acute coronary syndrome; NSTE-ACS, non-ST segment elevation acute coronary syndromes; RCS, retrospective cohort study; RCT, randomized controlled trial; BARC, Bleeding Academic Research Consortium.

### Risk of bias cochrane assessment tool version 2

**Figure 2:**
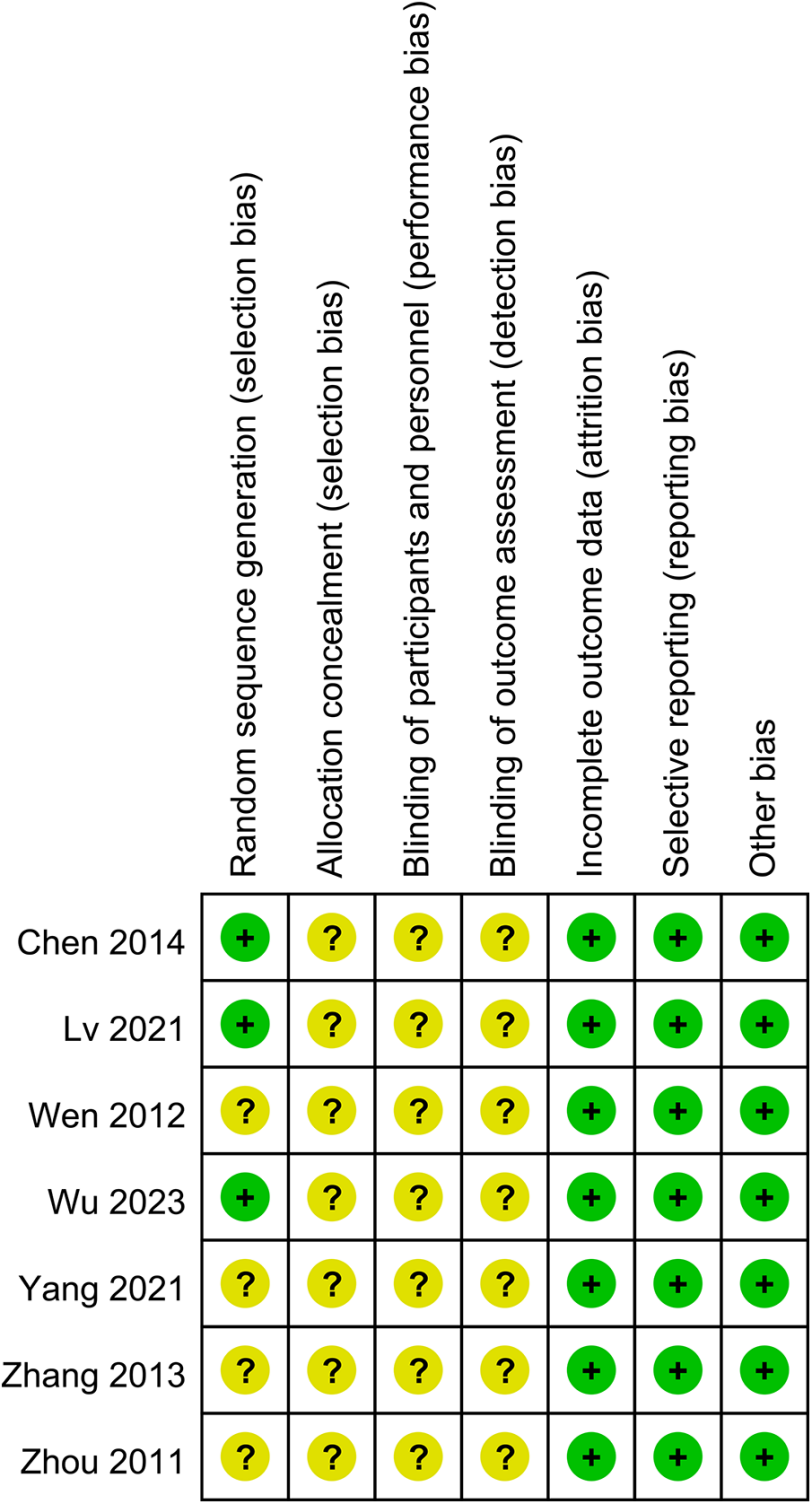
Risk of bias summary: review authors’ judgements about each risk of bias item for RCTs.

Seven RCTs deemed to carry some risk due to lack of specification regarding the specific follow-up methods and blinding procedures (15, 19–24). Overall, there was some risk of bias across studies in seven trails (Figure 2).

### Risk of bias ROBINS—I tool

One study was assessed as having a low risk of bias (18) and one study was classified as having a serious risk of bias (14). These assessments primarily stemmed from issues related to selection bias, intervention bias, measurement bias, and reporting bias. The details of risk bias were shown in Table 2.

**Table 2:** Risk of bias assessment with the ROBINS-I tool.

Study	Confounding bias	Selection bias	Classification bias	Intervention bias	Missing data bias	Measurement bias	Reporting bias	Overall risk of bias
Bai et al. (18)	Low	Low	Low	Low	Low	Low	Low	Low
Shi et al. (14)	Low	High	Low	Low	Low	Low	Low	High

### Adverse cardiovascular events

Five studies reported adverse cardiovascular events as a primary outcome (18, 19, 21, 23, 24). These events were categorized as recurrent angina, nonfatal myocardial infarction, and cardiac death.

### Recurrence of angina pectoris

**Figure 3:**
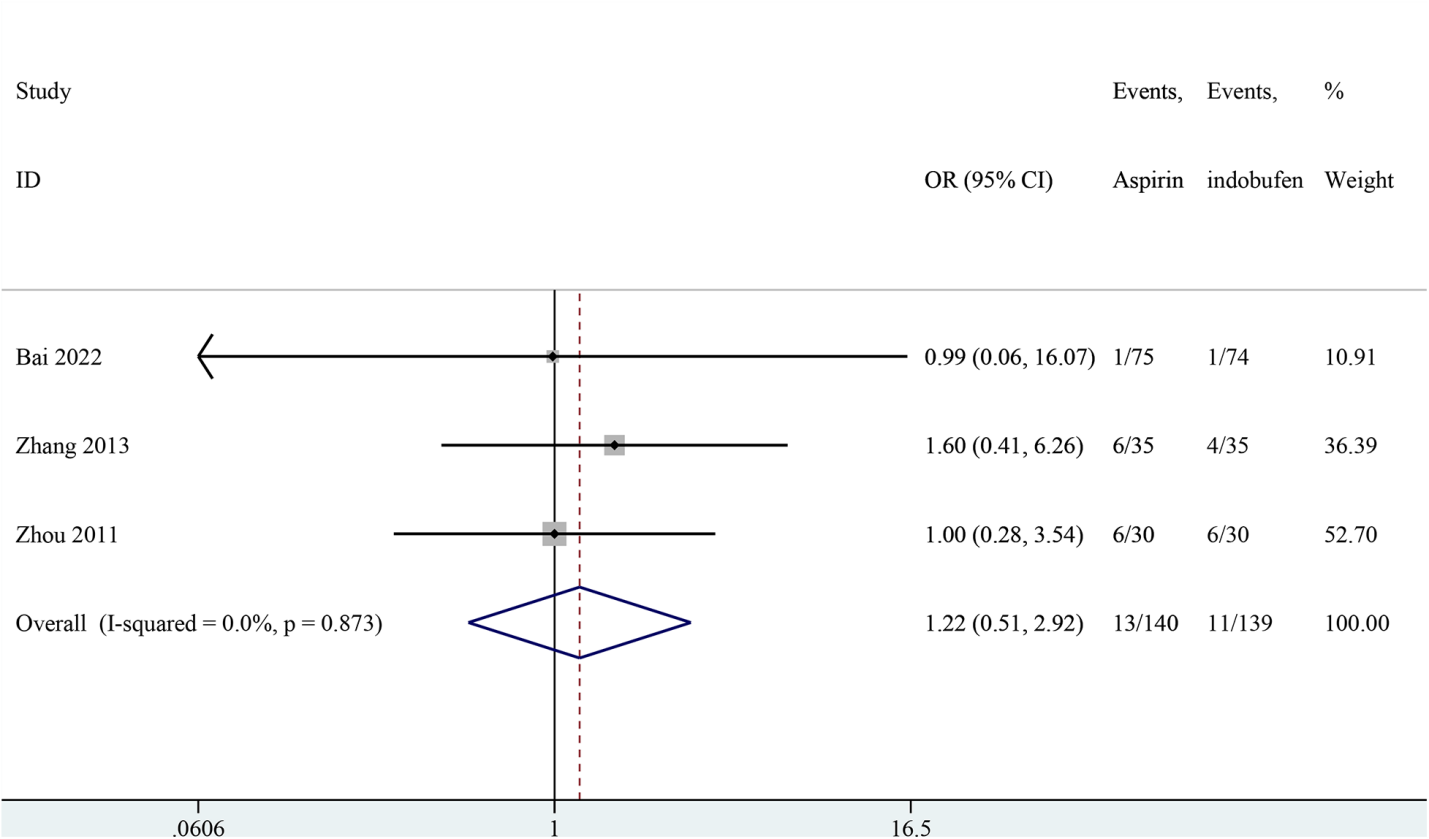
Forest plot of comparison: aspirin vs. indobufen; outcome: recurrence of angina pectoris.

Three studies addressed the recurrence of angina pectoris (18, 21, 24). The pooled results showed no significant difference in the recurrence rate of angina pectoris between the indobufen and aspirin group (OR: 1.22, 95% CI: 0.51–2.93, *I*^2^ = 0%, *P* = 0.659; Figure 3).

### Non-fatal myocardial infarction

**Figure 4:**
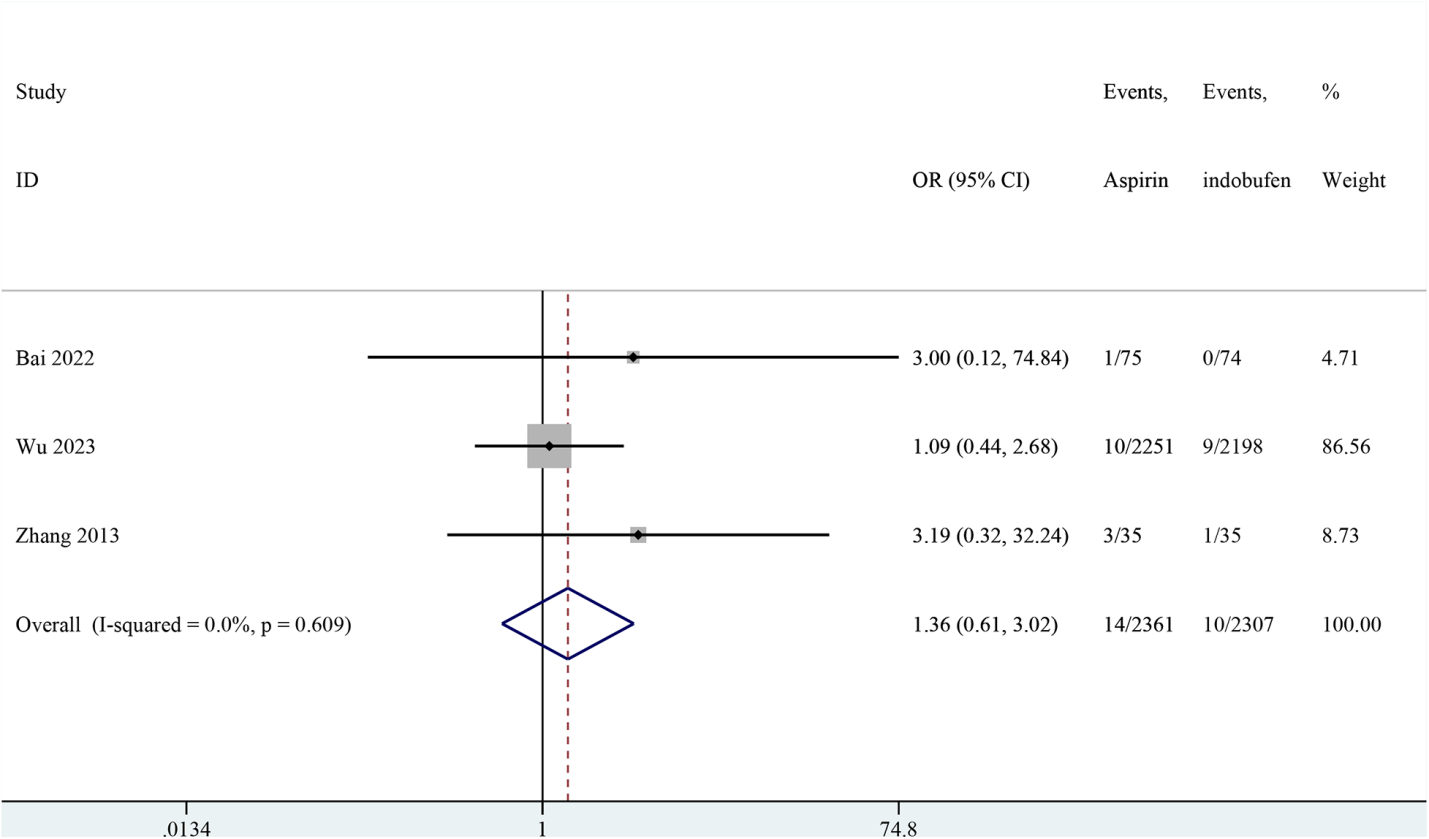
Forest plot of comparison: aspirin vs. indobufen; outcome: Non-fatal myocardial infarction.

Three studies reported non-fatal myocardial infarction (18, 19, 24). The pooled results indicated that no significant difference in the incidence of non-fatal myocardial infarction between the indobufen and aspirin group (OR: 1.36, 95% CI: 0.61–3.02, *I*^2^ = 0%, *P* = 0.451; Figure 4).

### Cardiovascular death

**Figure 5:**
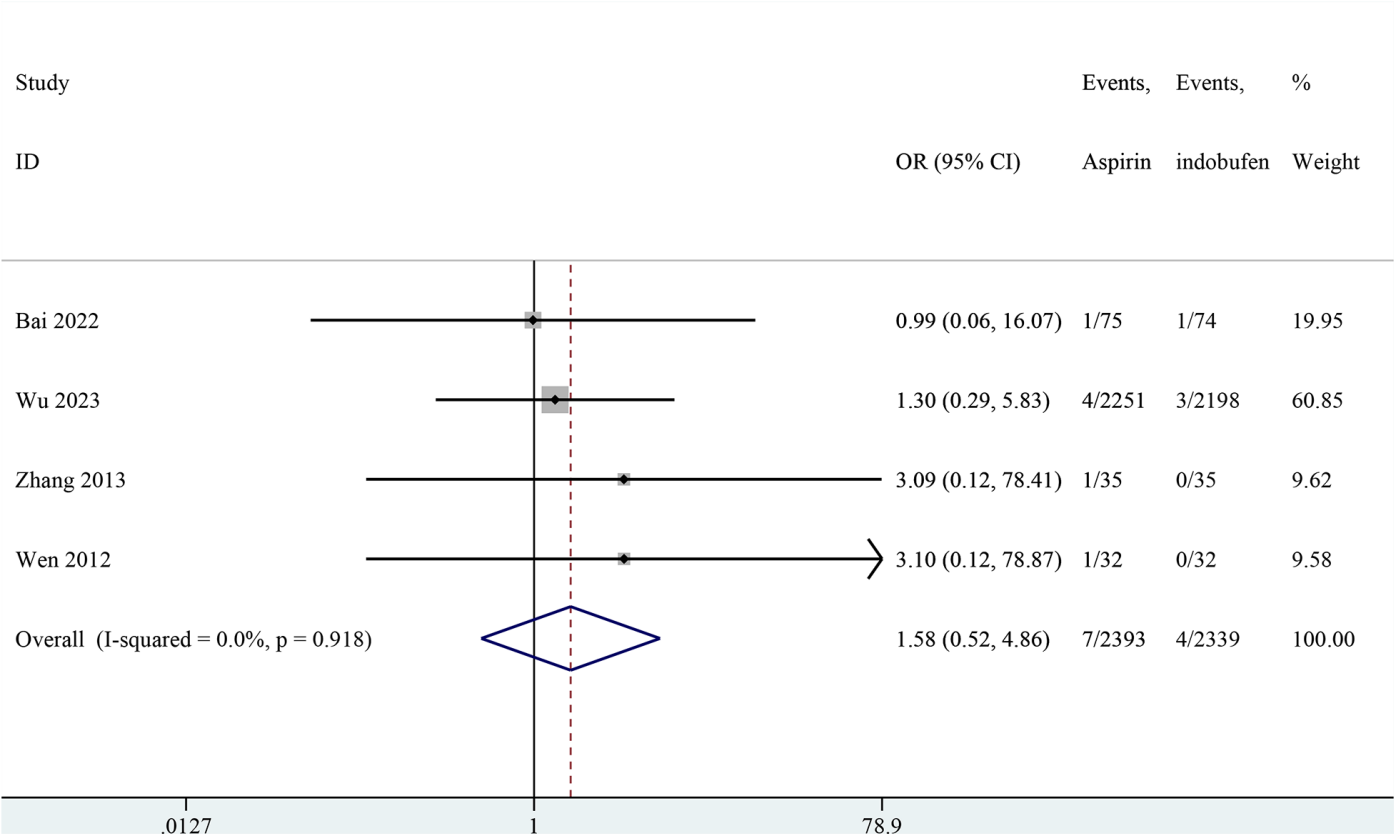
Forest plot of comparison: aspirin vs. indobufen; outcome: cardiovascular death.

Four studies included cardiovascular death (18, 19, 23, 24). The combined results showed no significant difference in cardiovascular death between the indobufen and aspirin group (OR: 1.58, 95% CI: 0.52–4.86, *I*^2^ = 0%, *P* = 0.422; Figure 5).

### Bleeding events

Nine studies reported on bleeding events (14, 15, 18–24). Bleeding events were classified according to academic research standards into minor bleeding (BARC grades 0–2), major bleeding (BARC grades 3–5) and any bleeding (BARC grade 0–5).

### Minor bleeding

**Figure 6:**
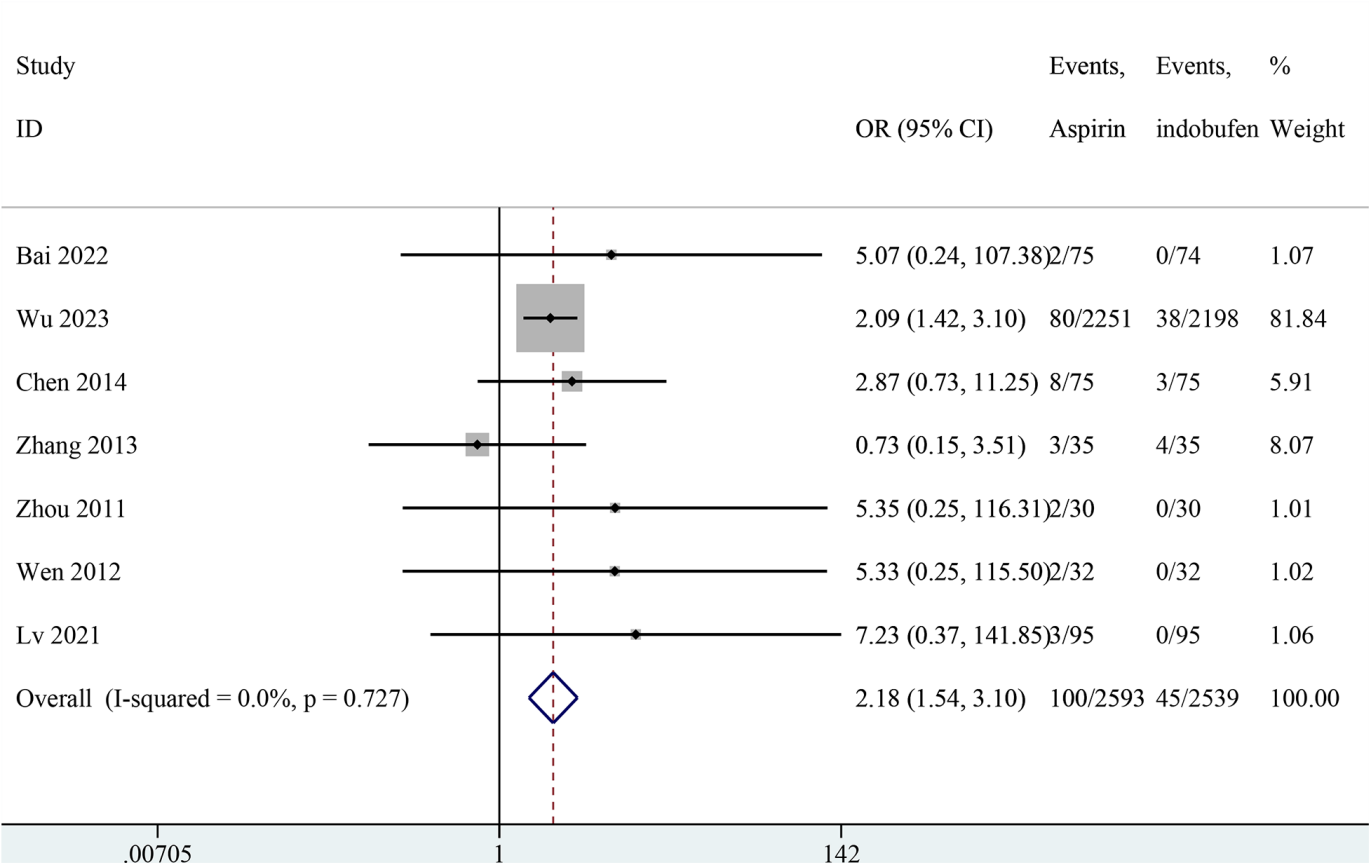
Forest plot of comparison: aspirin vs. indobufen; outcome: Minor bleeding.

Nine studies reported minor bleeding events (14, 15, 18–24). Two studies were excluded from the pooled meta-analysis results because both groups in these studies reported zero events (14, 15). The pooled results showed a significant reduction in minor bleeding events with indobufen compared to aspirin (OR: 2.18, 95% CI: 1.54–3.10, *I*^2^ = 0%, *P* < 0.001; Figure 6).

### Major bleeding

**Figure 7:**
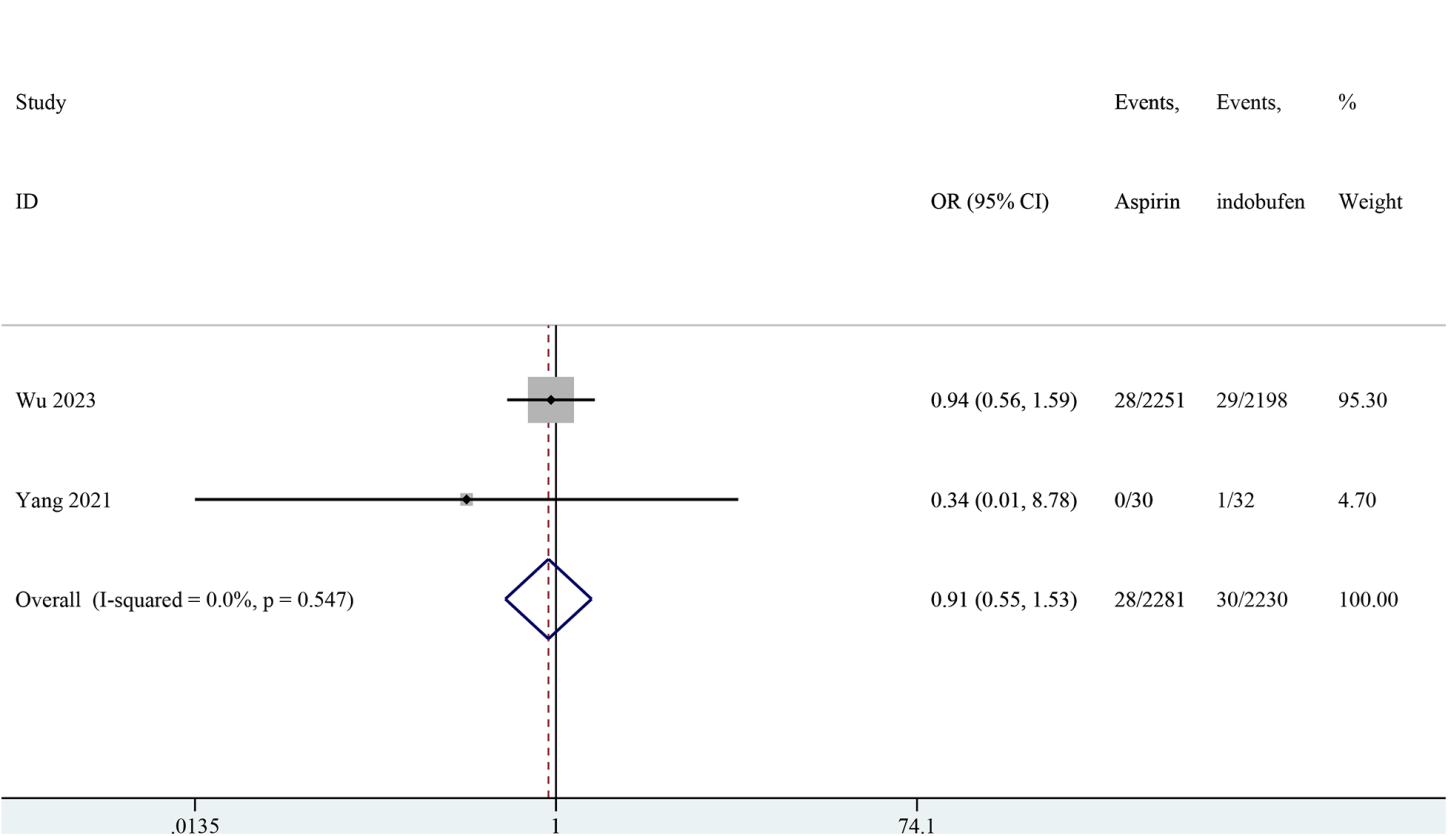
Forest plot of comparison: aspirin vs. indobufen; outcome: Major bleeding.

Two studies reported major bleeding events (15, 19). The pooled results showed no significant difference in major bleeding events between the indobufen and aspirin groups (OR: 0.91, 95% CI: 0.55–1.53, *I*^2^ = 0%, *P* = 0.732; Figure 7).

### Any bleeding

**Figure 8:**
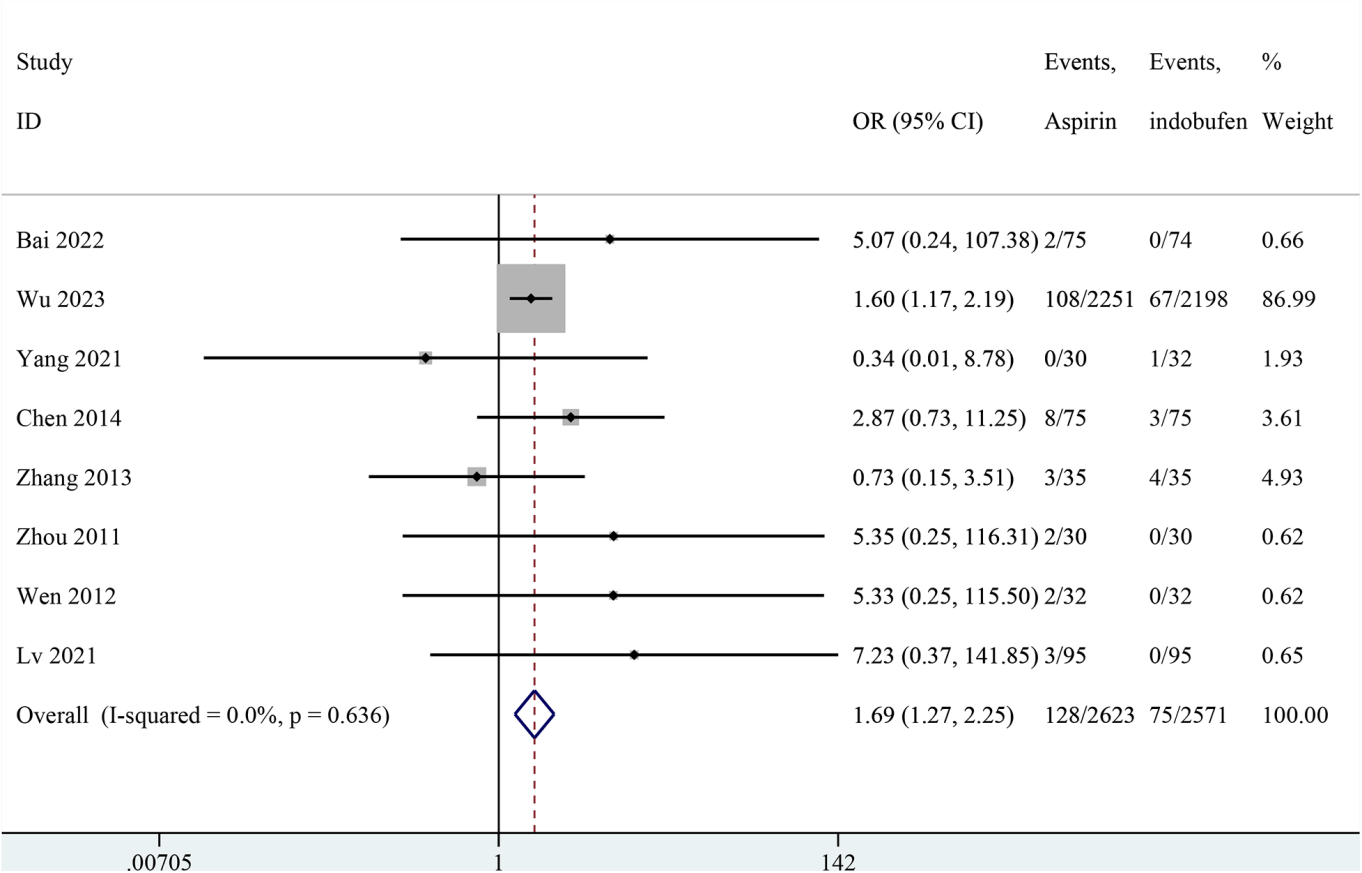
Forest plot of comparison: aspirin vs. indobufen; outcome: Any bleeding.

Nine studies reported any bleeding events (14, 15, 18–24). One study was excluded from the pooled meta-analysis results because both groups in these studies reported zero events (14). The pooled results showed a significant reduction in any bleeding events with indobufen compared to aspirin (OR: 1.69, 95% CI: 1.27–2.25, *I*^2^ = 0%, *P* < 0.001; Figure 8).

### Gastrointestinal adverse

**Figure 9:**
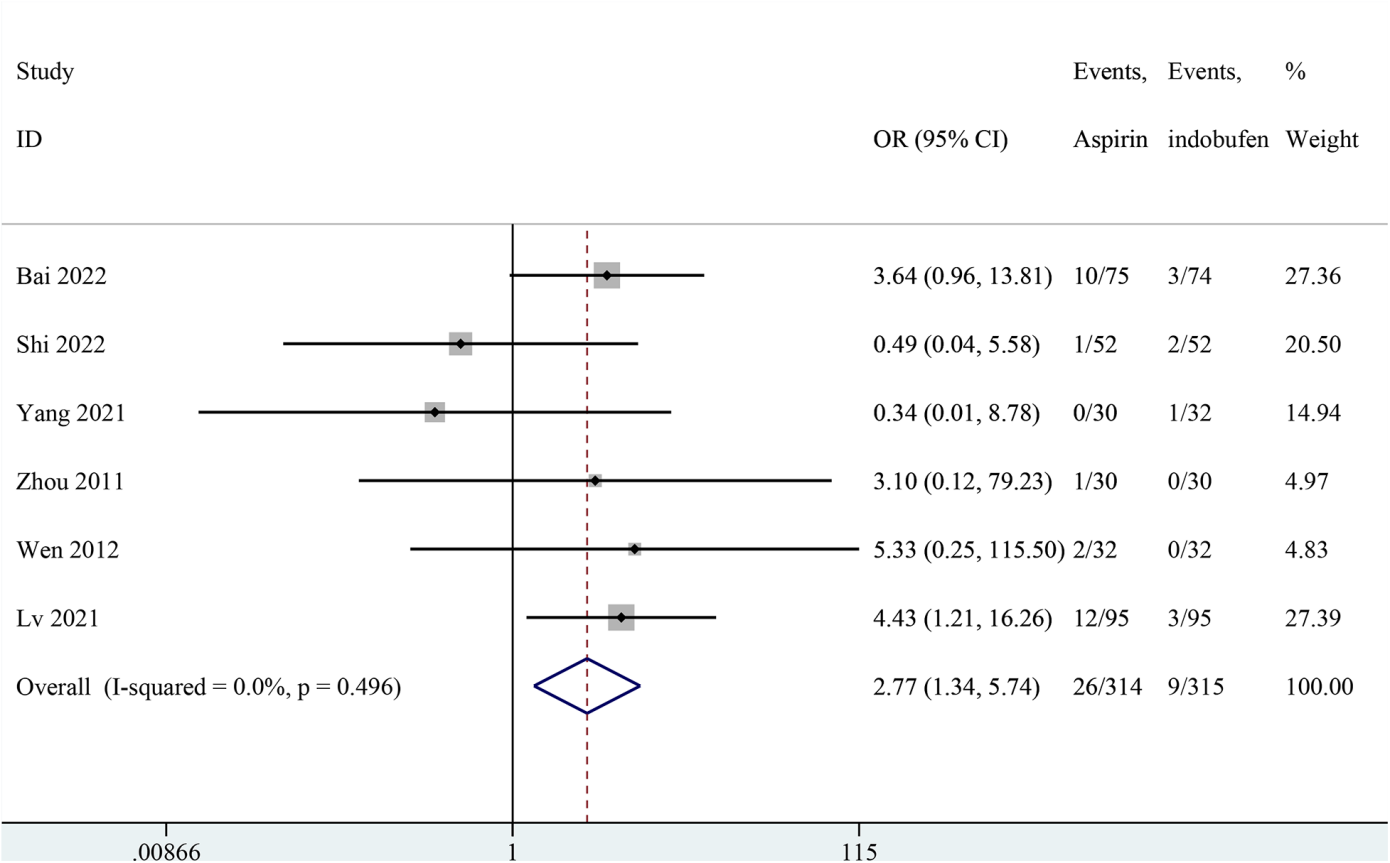
Forest plot of comparison: aspirin vs. indobufen; outcome: gastrointestinal adverse.

Six studies reported gastrointestinal adverse reactions (14, 15, 18, 21–23). The results showed that gastrointestinal reaction were significantly less frequent in the indobufen group compared to the aspirin group (OR: 2.77, 95% CI: 1.34–5.74, *I*^2^ = 0%, *P* = 0.006; Figure 9).

### Sensitivity analysis

No individual study had a significant impact on the results. The results of sensitivity analysis were shown in Supplementary Figure S1–S7.

### Publication bias

As fewer than ten trials were included, no publication bias assessment was performed by funnel plots.

## Discussion

Data from this study reaffirm that indobufen significantly reduces the incidence of minor bleeding events (BARC grades 0–2) and gastrointestinal adverse reactions, highlighting it's superior safety profile compared to aspirin. In terms of effectiveness, indobufen was comparable to aspirin regarding in the incidence of recurrent angina pectoris, myocardial infarction, and death from coronary heart disease. This meta-analysis included seven RCTs, all involving patients from China, providing a reference for current clinical practice, particularly for long-term antiplatelet monotherapy in Asian patients with a history of digestive tract diseases. There was no heterogeneity among the studies (*I*^2^ = 0), suggests a reliable conclusion. Additionally, indobufen significantly reduced the risk of any bleeding events compared to aspirin, consistent with previous studies. Therefore, the use of indobufen can alleviate concerns about gastrointestinal adverse effects associated with aspirin use. However, while the meta-analysis showed no heterogeneity (*I*^2^ = 0), suggesting consistency in the findings, it is essential to acknowledge the broader context of heterogeneity in the literature. Differences in study designs, ranging from randomized controlled trials to retrospective cohort studies, can introduce variability in outcomes due to differing methodologies. The patient populations also varied widely, including those undergoing procedures like coronary artery bypass grafting and percutaneous coronary intervention, each with different baseline characteristics such as age and sex distribution, which could influence the results. Treatment regimens also differed, with some studies combining indobufen or aspirin with clopidogrel, potentially leading to synergistic effects not seen in monotherapy. Moreover, the outcomes assessed, such as adverse cardiovascular events and gastrointestinal bleeding, were defined and reported inconsistently across studies. Despite these variations, the consistent finding that indobufen reduces gastrointestinal bleeding and adverse reactions compared to aspirin provides robust evidence for its clinical use, particularly for patients at high risk of bleeding or with aspirin intolerance.

Our meta-analysis confirms that indobufen significantly reduces gastrointestinal bleeding events compared to aspirin, aligning with guidelines that recommend indobufen for its lower incidence of gastrointestinal reactions and bleeding (2). This makes indobufen a viable option for patients with aspirin intolerance or those at high risk for bleeding and gastric ulcers, showing promising prospects for clinical application. For instance, studies have demonstrated that indobufen's efficacy is comparable to that of aspirin combined with dipyridamole after coronary artery bypass grafting (CABG). Furthermore, in preventing coronary restenosis post-percutaneous transluminal coronary intervention (PCI), indobufen combined with clopidogrel has shown to be safer with fewer side effects than aspirin combined with clopidogrel (23). Additionally, indobufen has proven effective and safe in preventing thromboembolic events in patients with non-rheumatic atrial fibrillation (25).

A broader meta-analysis on indobufen's efficacy and safety, either alone or in combination with other conventional drugs for ischemic cardiovascular and cerebrovascular diseases, found significantly lower incidences of bleeding, gastrointestinal reactions, and overall adverse drug events in the indobufen group. Indobufen also showed lower rates of bleeding and gastrointestinal reactions than other antiplatelet drugs and was significantly superior to placebo or no treatment in preventing thromboembolic events (26). Xu et al. systematically reviewed the efficacy and safety of indobufen in preventing cardiovascular and cerebrovascular events, finding no significant differences between indobufen and aspirin in preventing recurrent angina, nonfatal myocardial infarction, and cardiac death. However, total bleeding events and gastrointestinal reactions were less frequent with indobufen (27). Tian et al. evaluated indobufen's efficacy, safety, and cost-effectiveness compared to anticoagulants in patients with nonvalvular atrial fibrillation, including 72,599 patients across five studies. They found that indobufen reduced net clinical benefit events and clinically relevant non-major bleeding events, with no major bleeding events reported during the observation period (28).

In conclusion, while aspirin has a broader range of indications, indobufen is better tolerated and safer for inhibiting platelet aggregation. Economically, however, indobufen is less favorable. The average daily cost of indobufen (27.8 yuan/day) is significantly higher than that of aspirin (0.48 yuan/day). Despite its advantage, indobufen is not yet positioned to replace aspirin as the frontline treatment due to the extensive evidence-based support for aspirin in preventing and treating cardiovascular and cerebrovascular diseases. Indobufen remains a recognized alternative for patients intolerant to aspirin, but more extensive evidence is needed to challenge aspirin's primary status.

### Limitation

This study has several limitations. First, it included seven RCTs with high methodological quality evidence, one retrospective cohorts, and one open-label crossover study. RCTs are advantageous because they minimize bias and provide more robust causal inference by randomly assigning participants and controlling for various factors, making their conclusions more reliable. However, retrospective studies have inherent limitations due to the researchers' lack of control over data collection. The integrity and authenticity of records in these studies directly impact the reliability of the results, introducing significant bias. Open-label crossover studies are prone to ascertainment bias because researchers and participants are aware of the treatment assignment, which can influence the assessment of outcomes. For instance, researchers might subconsciously favor treatment plan A, leading to biased data collection for group A participants. researchers might subconsciously favor treatment plan A, leading to biased data collection for group A, participants.

Second, the study included patients with varying disease statuses, such as those with stable coronary artery disease, those who had undergone PCI, and those who had undergone CABG. Different studies evaluated treatment efficacy using diverse metrics, including the recanalization rate of the vascular bridge after aspirin and indobufen administration, coagulation function indicators such as FIB and D-dimer, plasma and urine Thromboxane B2 (TXB2) content, platelet aggregation rate (PAR), and platelet reactivity index (PRI-VASP, %). This variability limited the comparison of indicators in the final summary results, which foucused only on bleeding events, adverse gastrointestinal reactions, and cardiovascular events.

Thirdly, the dosing regimens in the included studies were inconsistent. Some studies compared aspirin or indobufen combined with clopidogrel, while others compared the efficacy and safety of aspirin and indobufen alone. The inability to account for the effect of concomitant clopidogrel use on gastrointestinal bleeding, gastrointestinal complaints, and cardiovascular event rates with introduced bias into the pooled analysis results.

Fourth, the literature search revealed that most studies comparing the safety and efficacy of indobufen and aspirin were conducted in China. This geographical limitation may introduce bias, as the results might not fully represent the global patient population. These limitations highlight the necessity for more diverse and comprehensive studies to provide clearer insights into the comparative safety and efficacy of indobufen and aspirin. While our findings are most applicable to Chinese or broader Asian populations, they offer valuable reference points for future research in different geographic and ethnic contexts. Future research should aim to include participants from various racial and ethnic backgrounds to validate these findings and ensure their global applicability. This approach will help to build a more comprehensive understanding of indobufen's benefits across diverse populations.

## Conclusion

The antiplatelet effect of indobufen is similar to that of aspirin, but indobufen offers some distinct advantages, including high selectivity and reversible binding to its receptor. Indobufen is associated with fewer gastrointestinal reactions and a lower risk of bleeding, making it an attractive option for patients who are intolerant to aspirin or have a high risk of bleeding and gastric ulcers. However, indobufen has a shorter history of use, and the evidence supporting its efficacy and safety is not as robust as that for aspirin. This limited scope and short history undermine the strength of its conclusions. Aspirin is more accessible and cost-effective due to its lower price. From a pharmacoeconomic perspective, aspirin remains the first choice for the primary and secondary prevention of cardiovascular and cerebrovascular diseases. Switching to indobufen or another antiplatelet agent should only be considered when aspirin is not tolerated or is contraindicated. As more large-scale clinical trials are conducted, the potential for indobufen to replace aspirin remains uncertain. In summary, while indobufen is a valuable alternative for specific patient populations, aspirin continues to be the preferred agent for most patients due to its well-established efficacy, safety profile, and cost-effectiveness.

## Data Availability

The original contributions presented in the study are included in the article/Supplementary Material, further inquiries can be directed to the corresponding author.
