# Supplementary material for: Safety and efficacy of aspirin and indobufen in the treatment of coronary heart disease: a systematic review and meta-analysis
Source: Front Cardiovasc Med. 2024 Aug 15;11:1412944. doi: 10.3389/fcvm.2024.1412944 (PMC11357911; doi:10.3389/fcvm.2024.1412944)
Supplement: Supplementary file 1 [file Datasheet1.pdf]

Supplementary Material

Safety and efficacy of aspirin and indobufen in the treatment of coronary heart disease: a systematic review and meta-analysis

Xiaochen Zhang[1†], Qiaoyan Yan[2†], Jiao Jiang[3†], Hua Luo[3], Yu Ren[2]

Correspondence: Hua Luo, 18732196660@163.com or Yu Ren, reny4147@enzemed.com

1. Supplementary Figures

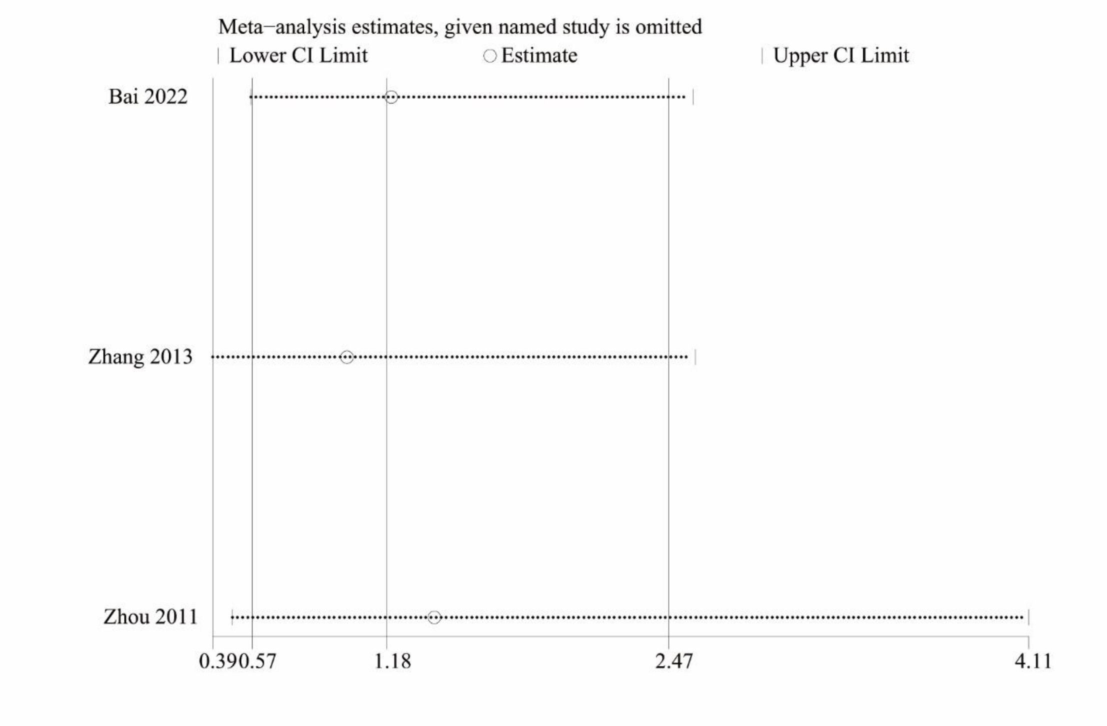

Supplementary Figure 1. Sensitivity analyses of the effects of recurrence of angina pectoris.

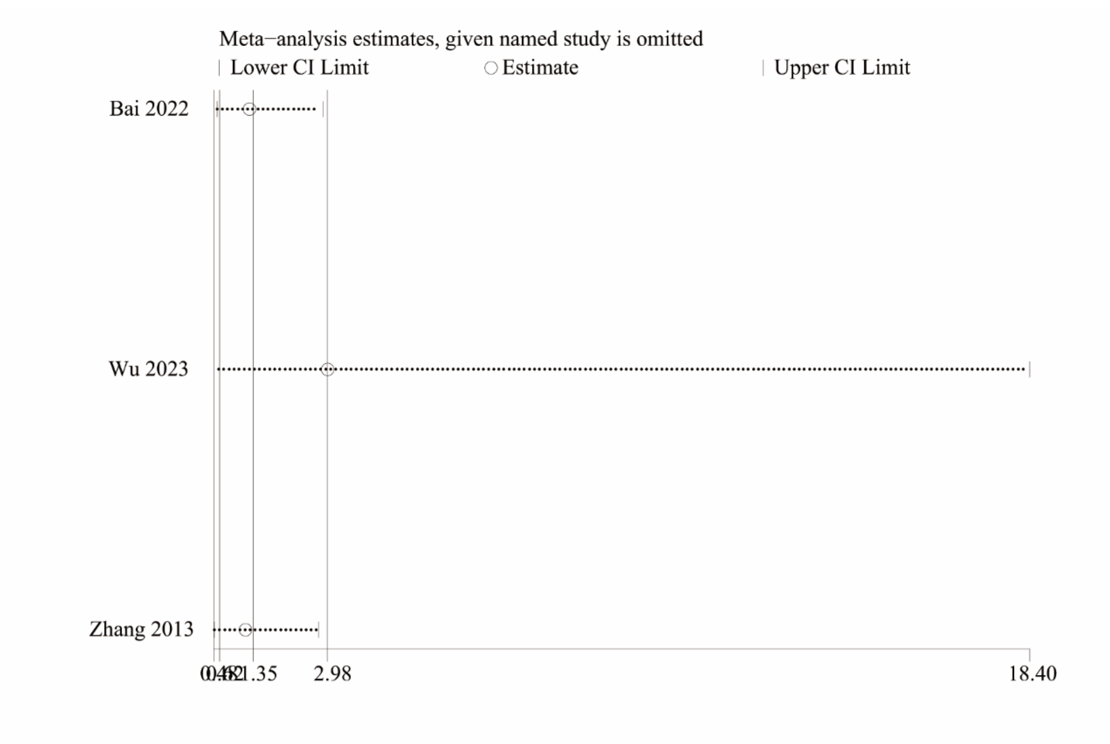

Supplementary Figure 2. Sensitivity analyses of the effects of non-fatal myocardial infarction.

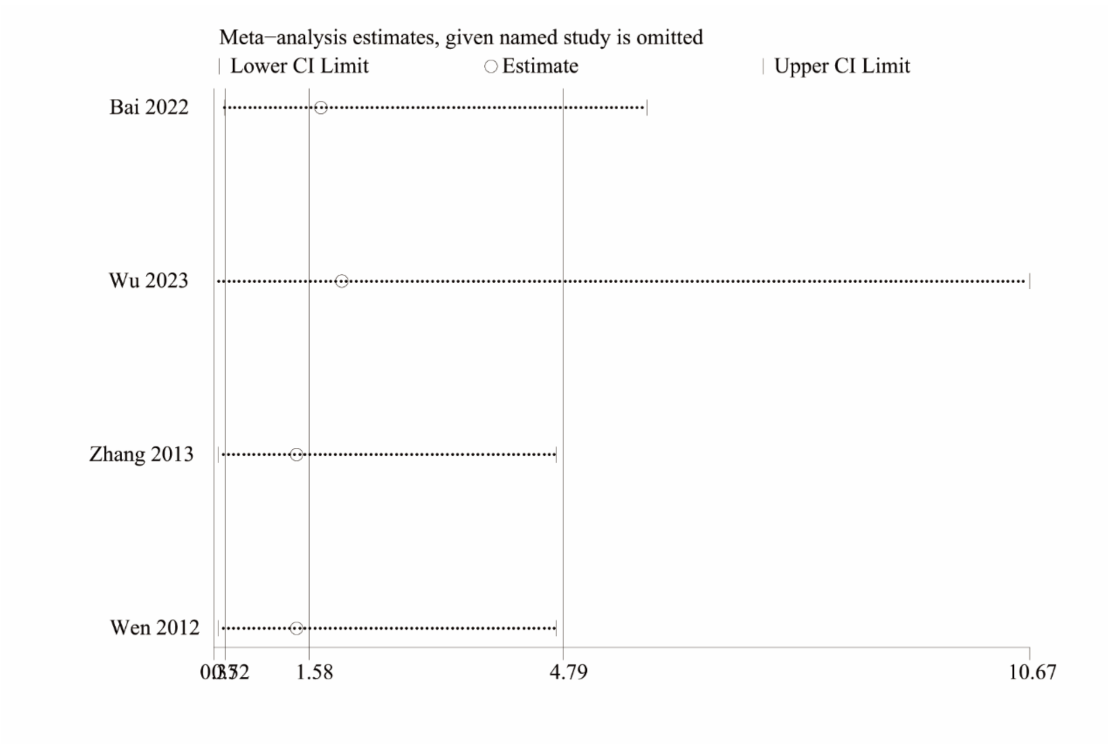

Supplementary Figure 3. Sensitivity analyses of the effects of cardiovascular death.

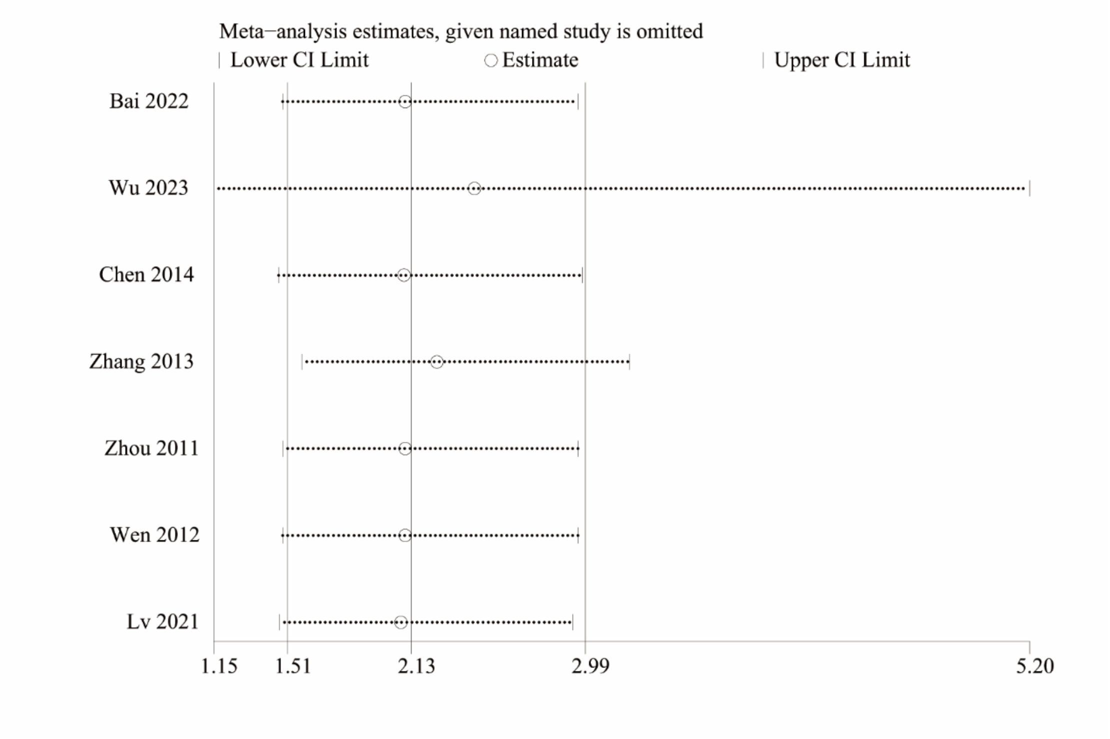

Supplementary Figure 4. Sensitivity analyses of the effects of minor bleeding.

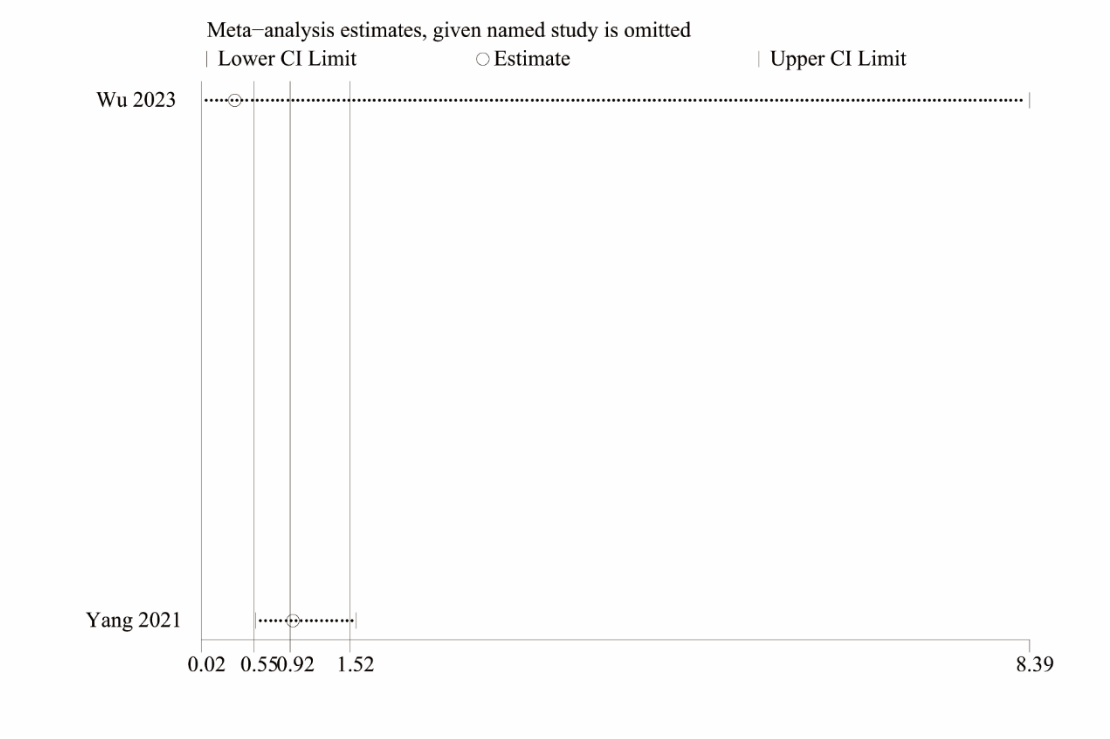

Supplementary Figure 5. Sensitivity analyses of the effects of major bleeding.

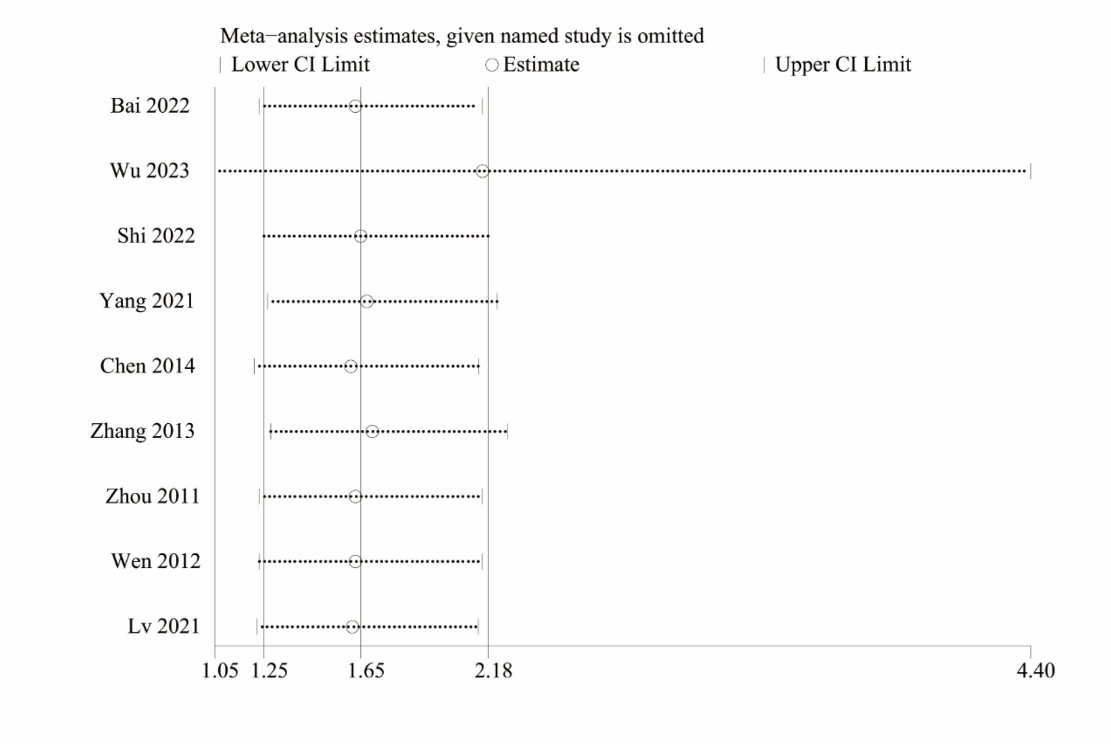

Supplementary Figure 6. Sensitivity analyses of the effects of any bleeding.

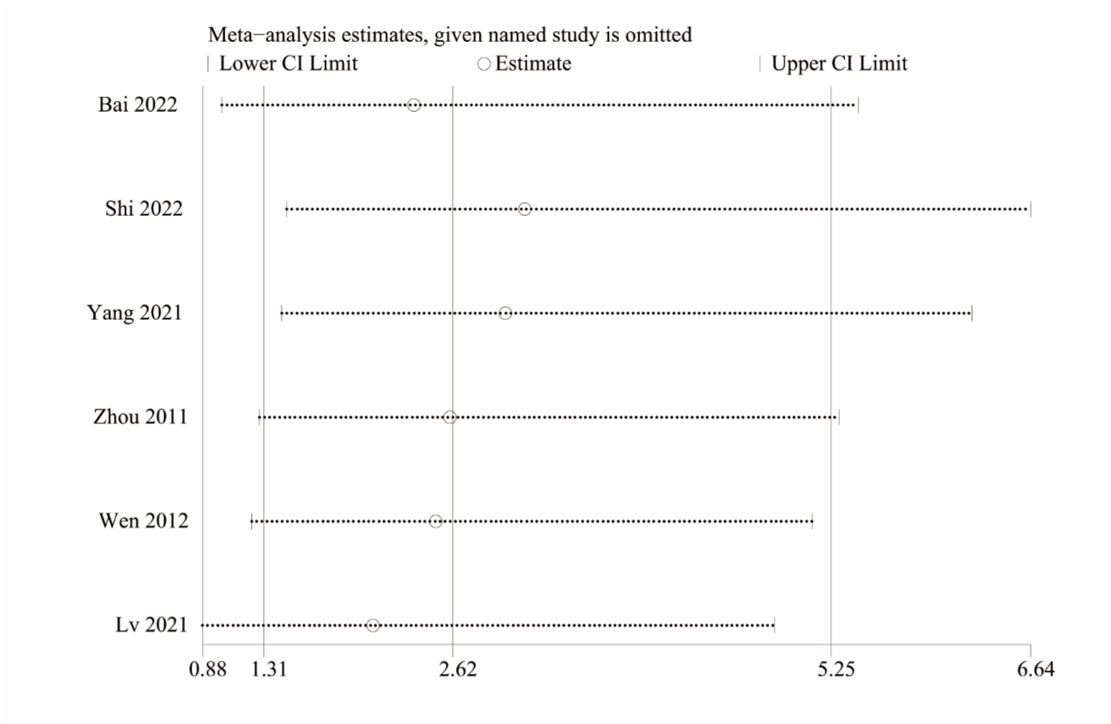

Supplementary Figure 7. Sensitivity analyses of the effects of gastrointestinal adverse.

2. Search strategy in databases

1) Search strategy in Pubmed

1. indobufen[Supplementary Concept]
2. indobufen[All Fields]
3. indobufene[All Fields]
4. 1 OR 2 OR 3
5. aspirin[MeSH Terms]
6. aspirin[All Fields]
7. aspirins[All Fields]
8. aspirin s[All Fields]
9. aspirine[All Fields]
10. acetylsalicylic[All Fields]
11. acid[All Fields]
12. acetylsalicylic acid[All Fields]
13. 10 AND 11
14. 5 OR 6 OR 7 OR 8 OR 9 OR 12 OR 13
15. acetylsalicyl[All Fields]
16. acetylsalicylate[All Fields]
17. acetylsalicylates[All Fields]
18. acetylsalicylic[All Fields]
19. 15 OR 16 OR 17 OR 18
20. aspirin[MeSH Terms]
21. aspirin[All Fields]
22. o acetylsalicylic acid[All Fields]
23. O-acetylsalicylate[All Fields]
24. 20 OR 21 OR 22 OR 23
25. anal sci adv[Journal]
26. asa[All Fields]
27. 25 OR 26
28. 14 OR 19 OR 24 OR 27
29. coronary disease[MeSH Terms]
30. coronary[All Fields]
31. disease[All Fields]
32. 30 AND 31
33. coronary disease[All Fields]
34. coronary[All Fields]
35. diseases[All Fields]
36. 34 AND 35
37. coronary diseases[All Fields]
38. coronary disease[MeSH Terms]
39. coronary[All Fields]
40. disease[All Fields]
41. 39 AND 40
42. coronary disease[All Fields]

43. coronary[All Fields]
44. heart[All Fields]
45. disease[All Fields]
46. 43 AND 44 AND 45
47. coronary heart disease[All Fields]
48. cancer health disparities[Journal]
49. chd[All Fields]
50. 48 OR 49
51. comput aided des[Journal]
52. cad[All Fields]
53. 51 OR 52
54. coronary artery disease[MeSH Terms]
55. coronary[All Fields]
56. artery[All Fields]
57. disease[All Fields]
58. 55 AND 56 AND 57
59. coronary artery disease[All Fields]
60. acute coronary syndrome[MeSH Terms]
61. acute[All Fields]
62. coronary[All Fields]
63. syndrome[All Fields]
64. 61 AND 62 AND 63
65. acute coronary syndrome[All Fields]
66. ann cardiothorac surg[Journal]
67. adv comm swallowing[Journal]
68. acs[All Fields]
69. 66 OR 67 OR 68
70. non st elevated myocardial infarction[MeSH Terms]
71. non st[All Fields]
72. elevated[All Fields]
73. myocardial[All Fields]
74. infarction[All Fields]
75. 71 AND 72 AND 73 AND 74
76. non st elevated myocardial infarction[All Fields]
77. nstemi[All Fields]
78. nstemis[All Fields]
79. 70 OR 75 OR 76 OR 77 OR 78
80. non-ST-segment[All Fields]
81. elevate[All Fields]
82. elevated[All Fields]
83. elevates[All Fields]
84. elevating[All Fields]
85. elevation[All Fields]
86. elevational[All Fields]

87. elevations[All Fields]
88. 81 OR 82 OR 83 OR 84 OR 85 OR 86 OR 87
89. myocardial infarction[MeSH Terms]
90. myocardial[All Fields]
91. infarction[All Fields]
92. 90 AND 91
93. st elevation myocardial infarction[MeSH Terms]
94. st[All Fields]
95. elevation[All Fields]
96. myocardial[All Fields]
97. infarction[All Fields]
98. 94 AND 95 AND 96 AND 97
99. st elevation myocardial infarction[All Fields]
100. stemi[All Fields]
101. stemis[All Fields]
102. 93 OR 98 OR 99 OR 100 OR 101
103. st elevation myocardial infarction[MeSH Terms]
104. st[All Fields]
105. elevation[All Fields]
106. myocardial[All Fields]
107. infarction[All Fields]
108. 104 AND 105 AND 106 AND 107
109. st elevation myocardial infarction[All Fields]
110. st[All Fields]
111. segment[All Fields]
112. elevation[All Fields]
113. myocardial[All Fields]
114. infarction[All Fields]
115. 110 AND 111 AND 112 AND 113 AND 114
116. st segment elevation myocardial infarction[All Fields]
117. angina, unstable[MeSH Terms]
118. angina[All Fields]
119. unstable[All Fields]
120. 118 AND 119
121. unstable angina[All Fields]
122. unstable[All Fields]
123. angina[All Fields]
124. 122 AND 123
125. angina, unstable[MeSH Terms]
126. angina[All Fields]
127. unstable[All Fields]
128. 126 AND 127
129. unstable angina[All Fields]
130. unstable[All Fields]

131. angina[All Fields]
132. pectoris[All Fields]
133. 130 AND 131 AND 132
134. unstable angina pectoris[All Fields]
135. urol ann[Journal]
136. ua[All Fields]
137. 135 OR 136
138. UAP[All Fields]
139. 117 OR 120 OR 121 OR 124 OR 125 OR 128 OR 129 OR 133 OR 134 OR 137 OR 138
140. 4 AND 28 AND 139

2) Search strategy in Embase

1. indobufen/exp
2. indobufen
3. 1 OR 2
4. aspirin/exp
5. aspirin
6. acetylsalicylic acid/exp
7. acetylsalicylic acid
8. acetylsalicylic
9. acid/exp
10. acid
11. 8 AND 9
12. 8 AND 10
13. o acetylsalicylic
14. acid/exp
15. acid
16. 13 AND 14
17. 13 AND 15
18. acetylsalicylate/exp
19. acetylsalicylate
20. o-acetylsalicylic acid
21. o acetylsalicylate
22. asa/exp
23. asa
24. 4 OR 5 OR 6 OR 7 OR 11 OR 12 OR 18 OR 19 OR 20 OR 16 OR 17 OR 21 OR 22 OR
25. coronary diseases
26. coronary
27. diseases/exp
28. diseases
29. 26 AND 27
30. 26 AND 28
31. coronary heart disease/exp
32. coronary heart disease
33. coronary
34. heart/exp
35. heart
36. disease/exp
37. disease
38. 33 AND 34
39. 33 AND 35
40. 36 AND 37
41. chd
42. cad/exp
43. cad

44. coronary artery disease/exp
45. coronary artery disease
46. coronary
47. artery/exp
48. artery
49. disease/exp
50. disease
51. 46 AND 47
52. 46 AND 48
53. 49 AND 50
54. acute coronary syndrome/exp
55. acute coronary syndrome
56. acute
57. coronary
58. syndrome/exp
59. syndrome
60. 56 AND 57 AND 58
61. 56 AND 57 AND 59
62. acs/exp
63. acs
64. nstemi/exp
65. nstemi
66. non–st-segment elevation myocardial infarction
67. non–st segment
68. elevation/exp
69. elevation
70. myocardial
71. infarction/exp
72. infarction
73. 67 AND 68
74. 67 AND 69
75. 70 AND 71
76. 70 AND 72
77. stemi/exp
78. stemi
79. st-segment elevation myocardial infarction/exp
80. st-segment elevation myocardial infarction
81. st segment/exp
82. st segment
83. elevation/exp
84. elevation
85. myocardial
86. infarction/exp
87. infarction

88. 82 AND 83
89. 82 AND 84
90. 85 AND 86
91. 85 AND 87
92. unstable angina/exp
93. unstable angina
94. unstable
95. angina/exp
96. angina
97. 94 AND 95
98. 94 AND 96
99. unstable angina pectoris/exp
100. unstable angina pectoris
101. unstable
102. angina/exp
103. angina
104. pectoris
105. 101 AND 102
106. 101 AND 103
107. 104 AND 103
108. ua
109. uap
110. 25 OR 29 OR 30 OR 31 OR 32 OR 38 OR 39 OR 40 OR 41 OR 42 OR 43 OR 44 OR 45 OR 51 OR 52 OR 53 OR 54 OR 55 OR 60 OR 61 OR 62 OR 63 OR 64 OR 65 OR 66 OR 73 OR 74 OR 75 OR 76 OR 77 OR 78 OR 79 OR 80 OR 88 OR 89 OR 90 OR 91 OR 92 OR 93 OR 97 OR 98 OR 99 OR 100 OR 105 OR 106 OR 107 OR 108 OR 109

3 AND 24 AND 110

3) Search strategy in Medline (via Web of Science)

1. Indobufen
2. Aspirin
3. acetylsalicylic acid
4. acetylsalicylate
5. O-acetylsalicylic acid
6. O-acetylsalicylate
7. ASA
8. 2 OR 3 OR 4 OR 5 OR 6 OR 7
9. Coronary Diseases
10. coronary heart disease
11. CHD
12. CAD
13. coronary artery disease
14. acute coronary syndrome
15. ACS
16. NSTEMI
17. non–ST-segment elevation myocardial infarction
18. STEMI
19. ST-segment elevation myocardial infarction
20. unstable angina
21. unstable angina pectoris
22. UA
23. UAP
24. 9 OR 10 OR 11 OR 12 OR 13 OR 14 OR 15 OR 16 OR 17 OR 18 OR 19 OR 20 OR 21 OR 22 OR 23
25. 1 AND 8 AND 24

4) Search strategy in Cochrane

Indobufen AND (Aspirin or acetylsalicylic acid or acetylsalicylate or O-acetylsalicylic acid or O-acetylsalicylate or ASA) AND (Coronary Diseases or coronary heart disease or CHD or CAD or coronary artery disease or acute coronary syndrome or ACS or NSTEMI or non–ST-segment elevation myocardial infarction or STEMI or ST-segment elevation myocardial infarction or unstable angina or unstable angina pectoris or UA or UAP) in Title Abstract Keyword
